# Do renewable energy and human capital facilitate the improvement of environmental quality in the United States? A new perspective on environmental issues with the load capacity factor

**DOI:** 10.1007/s11356-024-32331-z

**Published:** 2024-02-09

**Authors:** Serhat ÇAMKAYA, Abdulkerim KARAASLAN

**Affiliations:** 1 Department of Economics, Faculty of Economics and Administrative Sciences, Kafkas University, Merkez/KARS, Turkey; 2 Department of Econometrics, Faculty of Economics and Administrative Sciences, Atatürk University, Yakutiye/Erzurum, Turkey; 3 Master Araştırma Eğitim Ve Danışmanlık Hizmetleri Ltd. Şti. Ata Teknokent, Erzurum, Turkey

**Keywords:** Environmental quality, Load capacity factor, Renewable energy, Human capital, Augmented ARDL

## Abstract

Recently, countries have been making intensive efforts to alleviate the burden on the environment and to make environmental conditions sustainable. In this context, our study aims to investigate the long-term impact of renewable energy consumption (REC) and human capital (HC) by considering the load capacity factor (LCF). We also investigate the long-term impact of economic growth (Y) and non-renewable energy consumption (NREC) on the LCF. In this context, we analyze annual data for the U.S. for the period 1965–2018 using the newly developed augmented ARDL (AARDL) approach. The long-term empirical results show the following. i) Increases in Y negatively affect LCF and deteriorate environmental quality. ii) Increases in NREC negatively affect LCF and accelerate the deterioration of environmental quality. iii) REC has no significant impact on environmental quality. iv) Increases in HC support the improvement of environmental quality. The empirical results show that contrary to expectations, renewable energy consumption does not have a significant impact on environmental quality in the U.S., whereas human capital is an important factor in improving environmental quality. In this context, US policymakers should pave the way for more investment in eco-friendly renewable energy investments and human capital to establish sustainable environmental quality. Policymakers should also take steps to reduce the use of fossil fuels.

## Introduction

In today's world, environmental pollution is one of the most serious and worrying problems faced by countries. Our world has faced various environmental challenges such as excessive and unconscious use of natural resources, water scarcity, deforestation and consequent destruction of the habitats of various life forms. In particular, global warming as a result of increasing carbon dioxide (CO_2_) emissions is one of the biggest environmental problems we face (Haouas et al. 2023). Increasing global warming brings with it temperature increases. According to a study, it is predicted that temperatures may increase by 1.1 to 6.4 °C by the end of 2100. As a result of this situation, it is estimated that the sea level may rise by 16.5 to 53.8 cm by the end of 2100 by melting glaciers (Geneva 2013; Haouas et al. 2023). In recent times, global warming and the resulting climate change have been widely discussed among governments, scientists, and policymakers. In this direction, a number of steps have been taken to address and combat climate change, such as the Kyoto Protocol and the Paris Climate Agreement. Despite this, there has not yet been a significant global reduction in pollutant emissions (Saint Akadiri et al. 2020).

The deterioration of environmental quality due to the increase in environmental pollution affects all citizens of the world negatively and very seriously. Therefore, in recent years, there has been a considerable increase in the number of studies that identify the factors that cause this deterioration and develop various solutions to prevent deterioration and even improve environmental quality (Kartal 2023). Therefore, it is very important to identify the factors associated with climate change, which has the potential to cause global destruction and endanger the lives of all living things on the planet (Fareed et al. 2021; Adebayo 2022). Considering the existing literature, it is seen that the most important factor in the deterioration of environmental quality is the increase in non-renewable energy consumption(Adebayo 2022; Kartal et al. 2022, 2023). Therefore, the impact of non-renewable energy consumption on environmental quality has been investigated in empirical studies (e.g., Nathaniel and Khan 2020; Karaaslan and Çamkaya 2022). In addition, factors such as economic growth (e.g., Azam et al. 2023; Haouas et al. 2023), renewable energy consumption (e.g., Haouas et al. 2023; Ramzan et al. 2023) and human capital (e.g., Bano et al. 2018; Zafar et al. 2019; Jahanger 2022) have also been tested in the empirical literature.

The underlying factor behind global warming and the resulting climate change is thought to be the pollutant emissions released as a result of the burning of fossil fuels. With increasing industrialization, the use of fossil resources such as oil and coal is gradually increasing. This leads to an increase in pollutant emission levels globally (Haouas et al. 2023). In this context, the increase in the consumption of renewable energy sources is recognized as a good pathway towards the reduction of global CO_2_ emissions in comparison with non-renewable sources. The consumption of fossil resources leads to a further increase in global warming. Consequently, environmental degradation increases, and climate change occurs. For all these reasons, the international community is focusing on the use of the eco-friendly renewable energy (Pata 2021a). More widespread use of renewable energy can decrease the dependence on fossil resources and contribute to the reduction of pollutant emission levels. Moreover, wider adoption of renewable energy can also help prevent climatic and environmental degradation (Kirikkaleli and Adebayo 2021) Furthermore, an increase in the use of renewable energy can play a crucial role in addressing energy supply security concerns for countries.

Human capital stands out as another element that can play an important role in the reduction of pollutant emission levels. As pointed out by Bano et al. (2018), human capital affects individuals' capacity to manage environmental problems. Human capital increases the productivity of people by improving their industrial and production processes. Moreover, human capital can pave the way for countries to take steps to use environmentally friendly technologies and increase energy efficiency in areas where energy is used intensively such as industry, housing and transport (Zafar et al. 2019). Thus, the use of pollution-free technologies is encouraged. In addition, investigating the impact of human capital on environmental pollution may also help national economies to develop policies in line with sustainable economic development goals (Lan et al. 2012; Zafar et al. 2019). Therefore, human capital can be used as an effective tool for improving environmental quality and combating climate change.

**Fig. 1:**
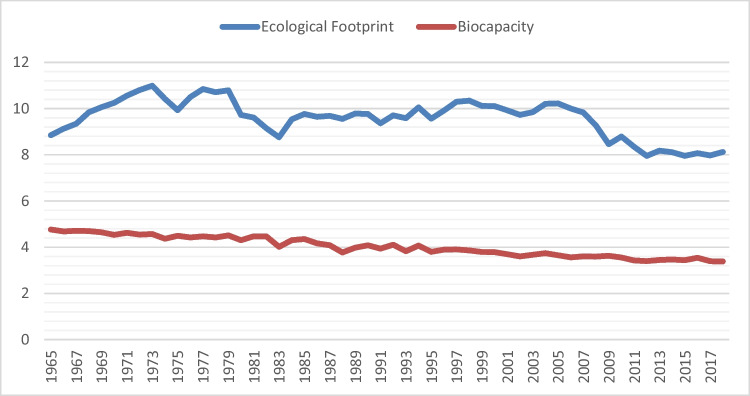
Environmental indicators in the USA. **Source:** Global Footprint Network (GFN 2023). **Notes:** The unit for biocapacity, and ecological footprint is the global hectares per person

**Fig. 2:**
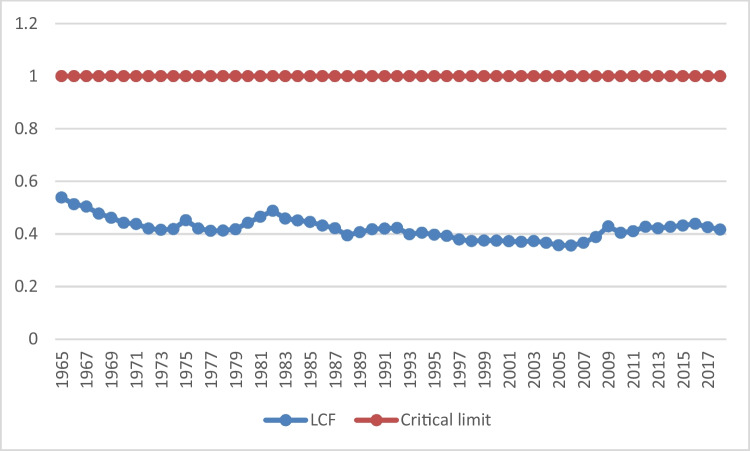
Environmental sustainability in the U.S. **Source:** Global Footprint Network (GFN 2023). **Notes:** The unit for LCF is the global hectares per person

In light of the above, the main objective of our study is to analyze the long-run impact of renewable energy consumption and human capital on LCF in the U.S. LCF reflects both the demand and supply side of the environment. Therefore, it is a more comprehensive environmental indicator than CO_2_ emissions and ecological footprint. From this point of view, we can determine whether renewable energy and human capital affect the demand side or supply side of the environment through the LCF and draw implications. In addition, we also investigate the long-run impact of economic growth and non-renewable energy consumption on the LCF. There are several reasons for taking the U.S. as a case study. The U.S. has achieved record economic growth in the last two decades. So much so that, according to the World Economic Outlook report prepared by the IMF (2022), the U.S. gross domestic product ranks first among 196 countries as of April 2022. Despite this economic success, the U.S. faces two very important environmental problems. The first of the problems is CO_2_ emission. As of 2022, the U.S. is the second most polluting country in the world after China with 4420.6 million tonnes (mt) of CO_2_ emissions (BP 2023). The second problem is that the U.S. relies heavily on fossil fuels in its energy consumption (WDI 2022). According to data published by BP (2023), the U.S. consumed 15.6 per cent of the world's total fossil fuels in 2021. In this respect, the U.S. is the second largest consumer of fossil fuels in the world (BP 2023). Moreover, the U.S. has the largest ecological footprint after China (GFN 2022). As can be seen in Fig. 1 below, the biocapacity in the U.S. has been lower than the ecological footprint over the years. This shows that the U.S. consumes more environmentally, but cannot replace this consumption. In Fig. 2, the course of LCF in the U.S. over the years is presented. Accordingly, the LCF has remained well below the sustainability threshold (i.e., 1) since 1965. Therefore, it is impossible to sustain the environmental conditions in the U.S. in terms of LCF. These situations can be presented as evidence that the U.S., one of the most polluting countries in the world, has a significant impact not only on its own environmental conditions but also on the environmental conditions of other countries in the world. Finally, the U.S. is in the category of "very high level of human development" according to the Human Development Report published by UNDP (2022). These issues constitute the main motivation behind why the U.S. is the subject of this study.

There is a limited number of studies investigating the impact of renewable energy consumption on LCF (Fareed et al. 2021; Pata 2021b; Pata and Samour 2022; Shang et al. 2022; Xu et al. 2022; Abdulmagid Basheer Agila et al. 2022). Furthermore, there is two study on the relationship between the LCF and human capital (Pata and Isik 2021; Pata et al. 2023). In addition, when the literature is examined, it is noteworthy that only three studies (Pata 2021b; Kartal et al. 2023; Pata et al. 2023) using LCF as a pollution indicator for the U.S. sample. However, there is no study investigating the simultaneous effects of renewable energy consumption and human capital on LCF. By investigating the simultaneous effects of human capital and renewable energy on LCF, we can see the effects of existing variables on LCF together. In this context, it can be emphasized which variable should be focused more on improving environmental quality and inferences can be made about which policies should be implemented. Therefore, there is a gap in the literature. Accordingly, this study, which focuses on the U.S., one of the most polluting countries in the world, can fill this gap by taking into account the importance of renewable energy and human capital in improving environmental quality for the U.S., which consumes fossil fuels intensively. In this context, our study makes the following contributions to the literature; i) To the best of our knowledge, the present study is the first to investigate the simultaneous impact of renewable energy consumption and human capital on the LCF for the U.S. ii) By considering the LCF as an indicator of environmental quality, the present study accounts for ecological footprint, which reflects the demand aspect of environmental quality, in conjunction with biological capacity, which reflects the supply aspect of environmental quality. This is done using the LCF, which addresses both indicators jointly. iii) The present study investigates the relationship between the variables using augmented ARDL (AARDL), a new time series method developed by McNown et al. (2018) and Sam et al. (2019). This approach allows obtaining more robust empirical results by testing the presence of a long-run relationship with three test statistics. In addition, the AARDL model is superior to the ARDL approach as it has a statistic for independent variables that can be used even when the dependent variable is I(0). Furthermore, the robustness check of the AARDL model was performed using FMOLS, DOLS and CCR methods. These approaches allow obtaining robust estimates by taking into account the endogeneity in the independent variables as well as possible autocorrelation and variance problems (Yahyaoui and Bouchoucha 2021). Therefore, we utilised these methods in our study. iv) Numerous developed and particularly developing countries around the world follow the policies of the U.S. Therefore, proposing a solution to the environmental problems faced by the U.S. becomes very important. In this context, the present study offers a set of policy recommendations for both developed and developing countries through an investigation of the impact of REC and HC for improving environmental quality in the case of the U.S.

The remainder of the study was organized as follows: “Literature review” section presents the literature review. “Research methodology” sections and “Empirical results and discussion” present the research methodology and empirical results and discussion, respectively. “Discussion” section concludes the study by providing conclusions and recommendations.

## Literature review

In this section, where the literature review is given, the literature is divided into three parts. In the first part, the theoretical literature is mentioned. In the second part, empirical literature is included. In the third and last part, the literature review is completed by mentioning the studies specific to the U.S.

### Theoretical literature

Simon Kuznets (1955) analysed the relationship between income and income distribution for the first time. Kuznets states that in the first stage of development in developing countries, a deterioration in income distribution will occur with increasing growth, but after a certain turning point, increasing growth will correct the deterioration in income distribution. This view is referred to in the literature as the Kuznets hypothesis. This hypothesis is called "Kuznets Curve" because it assumes an inverted-U shaped relationship between income distribution and economic growth. This hypothesis of Kuznets has also guided the studies on environmental quality. With the pioneering work of Grossman and Krueger (1991, 1995), a new hypothesis called "Environmental Kuznets Curve (EKC) Hypothesis" was put forward by replacing the income distribution axis in the original Kuznets curve with pollution.

The EKC hypothesis aims to examine the long-term relationship between environmental degradation and economic growth. As economic growth increases, in the take-off phase, the amount of waste generation increases as the rate of resource depletion exceeds the rate of resource renewal, and toxicity increases accordingly. At higher levels of development, there is increased environmental awareness, implementation of environmental regulations, increased technological infrastructure and structural changes towards knowledge-intensive industries and services with increased environmental expenditure. This results in stabilization and gradual reduction of environmental degradation. It is hypothesized that as income increases beyond the turning point of the EKC, environmental quality will start to improve. The EKC hypothesis can therefore be taken as a natural depiction of the transition from a clean agricultural economy to a polluting industrial economy and back to a clean service economy (Dinda 2004). With the EKC hypothesis, interest in environmental issues has increased and many studies on environmental issues have been and continue to be carried out in the literature.

In the literature, it is observed that CO_2_ emission is frequently used as a proxy variable in the measurement of environmental degradation. However, this variable is a weak indicator as it does not cover all environmental indicators such as soil, mining, and forestry (Ulucak and Apergis 2018). In order to address this shortcoming, ecological footprint (EF) began to be used for measuring environmental pollution. In addition to demonstrating the impact of human-based consumption on the environment, EF also compares the biosphere's rate of regeneration with anthropogenic consumption (Rees 1992). Although EF possesses certain advantages compared to CO_2_ emissions, it only encompasses the demand aspect of environmental degradation and disregards the supply, i.e. the biocapacity (BC) aspect. At this point, an indicator used in the measurement of environmental degradation has emerged, reflecting both the demand and supply aspects of the environment (Pata and Samour 2022). This indicator is referred to as the load capacity factor and measures the extent to which a region or country can sustain its population based on its existing way of life (Siche et al. 2010). The load capacity factor is calculated through dividing BC by EF (BC/EF). In the load capacity factor, the limit of sustainability is 1. If load capacity factor < 1, environmental conditions are regarded as unsustainable, whereas if load capacity factor > 1, environmental conditions are considered sustainable (Siche et al. 2010; Pata 2021b). Therefore, we used LCF to measure environmental quality in our study.

### Empirical literature

In this section of the study, the empirical literature on economic growth, non-renewable energy consumption, renewable energy consumption and human capital, which have an impact on environmental pollution, is briefly presented. In this context, the relationship between environmental pollution indicators and economic growth, non-renewable energy consumption, renewable energy consumption and human capital is discussed.

At the beginning of the literature review, previous studies examining the relationship between environmental pollution indicators and economic growth were included. Studies using CO_2_ emissions as the dependent variable found that CO_2_ emissions increased in parallel with economic growth (Apergis and Payne 2010; Shahbaz et al. 2014, 2021; Solarin et al. 2017; Raggad 2020; Omri and Saidi 2022). In addition to these studies, Khan and Ozturk (2020)for 17 Asian countries, Khan et al. (2022) for the Commonwealth of Independent States and Azam et al. (2023) and Haouas et al. (2023) for MENA countries have also obtained similar findings. There are also studies using ecological footprint as the dependent variable and reporting ecological footprint increases in parallel with economic growth (Al-mulali et al. 2015; Mrabet and Alsamara 2017; Baloch et al. 2019; Ahmed et al. 2021). Finally, other studies using the load capacity factor as the dependent variable found that increases in economic growth negatively affect the load capacity factor (Fareed et al. 2021; Shang et al. 2022; Abdulmagid Basheer Agila et al. 2022).

In the second phase of the literature review, studies examining the impact of non-renewable energy consumption on environmental pollution were included. Previous studies such as Shafiei and Salim (2014), Dogan and Seker (2016), Bekun et al. (2019), Chen et al. (2019), and Karaaslan and Çamkaya (2022) revealed that increases in non-renewable energy consumption lead to increases in CO_2_ emissions. Similarly, studies such as Alola et al. (2019), Destek and Sinha (2020), Nathaniel and Khan (2020), Ansari et al. (2022), and Dogan et al. (2022) found that non-renewable energy consumption has a positive effect on ecological footprint. Finally, albeit limited in number, there are also studies reporting that non-renewable energy consumption has a negative impact on load capacity factor (Fareed et al. 2021; Awosusi et al. 2022; Xu et al. 2022; Adebayo 2022).

In the third phase, previous studies examining the environmental impact of renewable energy consumption are discussed. Zoundi (2017) found that REC has a negative effect on CO_2_ emissions. Studies such as Sinha and Shahbaz (2018), Waheed et al. (2018), Mahmood et al. (2019), Saidi and Omri (2020), Chen et al. (2022), Liguo et al. (2022), Baloch and Danish (2022), and Omri and Saidi (2022) also obtained results confirming the negative relationship between the two variables. Danish et al. (2020) found that renewable energy consumption has a negative effect on ecological footprint. Usman and Makhdum (2021) and Mujtaba et al. (2022) obtained similar findings. Fareed et al. (2021), and Shang et al. (2022) concluded that renewable energy consumption has a negative effect on load capacity factor. However, in the study conducted by Menyah and Al-Mulali et al. (2015) for Vietnam, Khoshnevis Yazdi and Shakouri (2018) for Germany, and Pata (2018) for Turkey, it was determined that renewable energy consumption had no significant impact on CO_2_ emissions. Similarly, in the studies carried out by Nathaniel and Khan (2020) for ASEAN countries, Pata and Caglar (2021) for China, and Çakmak and Acar (2022) for the 8 countries with the highest oil production, it was shown that renewable energy consumption had no significant effect on ecological footprint. Pata and Samour (2022), on the other hand, found no significant relationship between load capacity factor and renewable energy consumption.

Finally, studies examining the impact of human capital on the environment were compiled. Bano et al. (2018), Yao et al. (2020), Rahman et al. (2021) and Jahanger (2022) found that human capital had a negative impact on CO_2_ emissions while Ahmed et al. (2020), Nathaniel et al. (2021) and Pata et al. (2021) reported that human capital had a negative effect on ecological footprint and Pata and Isik (2021) revealed that human capital had a positive effect on the load capacity factor.

### Literature on the U.S.

As for the U.S.-specific literature, Menyah and Wolde-Rufael (2010) tested the effect of renewable energy consumption on CO_2_ emissions using data from 1960–2007. Empirical findings show that renewable energy consumption has not reached a level that can reduce pollution. Baek (2016) applied annual time series data for the period 1960–2010 and ARDL method and found that renewable energy reduces CO_2_ emissions only in the short run. In addition, the study also found that economic growth increases environmental pollution in the long run. Dogan and Ozturk (2017) examined the effect of economic growth, renewable and non-renewable energy consumption on CO_2_ emissions for the period 1980–2014 using unit root with structural breaks, cointegration and estimation methods. The results show that growth and non-renewable energy consumption increase environmental pollution, while non-renewable energy consumption decreases it. Similar findings were obtained from Khan and Hou (2021), Pata (2021c), Liguo et al. (2022), Hossain et al. (2023) and Yi et al. (2023).

Zafar et al. (2019), using the ARDL procedure, found that economic growth has a positive effect on ecological footprint, but human capital has a negative effect. Usman et al. (2020) similarly used the ARDL method and examined the effect of renewable energy consumption and economic growth on ecological footprint. ARDL findings show that renewable energy consumption increases environmental quality by reducing the ecological footprint, while economic growth leads to the opposite result. Similarly, Ramzan et al. (2023) also examined the impact of renewable energy consumption and economic growth on ecological footprint and found that they have a negative and positive relationship on ecological footprint, respectively.

Pata (2021b) tested the long-run effect of renewable energy and economic growth on the load capacity factor in the U.S. and Japan. The empirical findings of the study show that renewable energy increases environmental quality by increasing the load capacity factor in the U.S., but renewable energy has no significant effect on environmental quality in Japan. The findings also reveal that economic growth decreases the load capacity factor in both countries. In this context, economic growth deteriorates environmental quality. Similar studies were conducted by Kartal et al. (2023) and Pata et al. (2023). The results of both studies confirm that clean energy increases the load capacity factor and thus environmental quality, while economic growth produces the opposite result.

When the existing empirical literature is examined, we see that CO_2_ emission and ecological footprint are used extensively to measure environmental pollution. However, it is noteworthy that the load capacity factor is used in only a few studies compared to other pollution indicators. In addition, there is only one study in the empirical literature that analyses the human impact on the load capacity factor. When the U.S. specific literature is analyzed, it is observed that a similar situation emerges. Therefore, it is noticeable that there is a gap in the literature. We try to fill this gap in the literature by analyzing the effect of human capital on load capacity. Moreover, it is observed that conventional unit root and cointegration methods are generally used to test empirical models in the existing literature. In our study, we differentiate ourselves from the existing literature by using unit root tests with structural breaks and the recently developed AARDL approach, which is very popular and provides strong empirical evidence. Thus, we aim to contribute to the existing literature.

## Research methodology

### Data

In the present study, the long-run effects of economic growth, non-renewable energy consumption, renewable energy consumption and human capital on the load capacity factor for the U.S. are investigated using annual time series data for the period from 1965 to 2018. The reason why 1965 was chosen as the starting year in the study is that the data for non-renewable energy consumption and renewable energy consumption variables are available up to this date. The reason for choosing 2018 as the end year is that the data for the human capital variable is not available after this date. Table 1 shows the details of the variables used.

**Table 1:** Variables

Symbol	Variables description	Unit	Source
LCF	Load capacity factor	Biocapacity/ecological footprint (gha)	GFN (2022)
Y	Gross domestic product	Constant 2015 US $ (per capita)	WDI (2022)
NREC	Non-renewable energy consumption	Fossil fuels per capita (kWh)	OWD (2022)
REC	Renewable energy consumption	Renewables per capita (kWh)	OWD (2022)
HC	Human capital	Index of human capital (per capita)	Penn world table version 10.0 (2015)

The LCF variable we used in our study is used in Pata and Isik (2021) and Pata et al. (2023), the Y variable is used in Apergis and Payne (2010), Shahbaz et al. (2014), Raggad (2020) and Omri and Saidi (2022), NREC and REC variables are used in Destek and Sinha (2020), Nathaniel et al. (2021), Ansari et al. (2022), Dogan et al. (2022) and Sinha and Shahbaz (2018), Mahmood et al. (2019), Saidi and Omri (2020), Khan and Hou (2021), Baloch and Danish (2022) and finally the HC variable was selected by considering the studies of Bano et al. (2018), Zafar et al. (2019), Yao et al. (2020), Rahman et al. (2021) and Jahanger (2022).

### Model

Human capital is defined by Bano et al. (2018) as the most important of all inputs used in the production phase. As stated by Zafar et al. (2019), human capital includes the level of knowledge and experience, experience and skills, and education of people in an economy. In this context, the output produced in an economy with a high level of human capital is likely to be high value-added, in other words, high-tech output. These high-tech products are also likely to have the potential to improve environmental quality. When the studies on the impact of human capital on environmental quality in the U.S. are analyzed, it is seen that these studies are both very few and yield mixed results. For example, Dedeoğlu et al. (2021) found that human capital has no significant effect on environmental quality, while Zafar et al. (2019) and Pata et al. (2023) found that human capital is important in improving environmental quality.

The existing literature shows that renewable energy is an important instrument to improve environmental quality (Zoundi 2017; Mahmood et al. 2019; Baloch and Danish 2022; Liguo et al. 2022; Hossain et al. 2023; Pata et al. 2023). On the contrary, increasing non-renewable energy consumption will negatively affect environmental quality. This is because the increase in fossil fuel consumption will lead to the release of more polluting gases into the environment. As a natural consequence of this situation, environmental quality will decrease. However, increasing the use of environmentally friendly renewable energy sources will help to improve environmental quality by preventing the release of pollutant gases to nature. Finally, as noted by Pata et al. (2023), despite the high income level of the U.S. economy, its economic growth still increases pollution and deteriorates environmental quality.

In line with the foregoing, the impact of human capital, renewable and non-renewable energy consumption and economic growth on environmental quality in the U.S. is included in the research model.

The model used for the empirical analysis in this study is based on Menyah and Wolde-Rufael (2010), Zafar et al. (2019) and Yi et al. (2023). In these studies, CO_2_ emission and ecological footprint were used as environmental pollution indicators and dependent variables. Unlike these studies, the present study uses load capacity factor as an environmental pollution indicator. In this study, the natural logarithm of all variables was taken to avoid heteroscedasticity and to obtain the elasticities of the variables using a double logarithmic model. The empirical model of the study is as follows:

1 $${\text{ln}}LC{F}_{t}={\gamma }_{0}+{\gamma }_{1}{\text{ln}}Y+{\gamma }_{2}{\text{ln}}NREC+{\gamma }_{3}{\text{ln}}REC+{\gamma }_{4}{\text{ln}}HC+{e}_{t}$$

here; ln = logarithmic term, t = time, ${\gamma }_{0}$= constant term, ${\gamma }_{1}$, ${\gamma }_{2}$, ${\gamma }_{3}$, ${\gamma }_{4}$= long-run elasticities of Y, NREC, REC and HC on LCF, respectively, and ${e}_{t}$= error term.

The sign of ${\gamma }_{1}$ is negative when the scale effect is dominant and positive when the technical effect is dominant. This depends on countries' level of development (Pata and Balsalobre-Lorente 2022). The sign of ${\gamma }_{2}$ is expected to be positive as the use of fossil fuels increases the negative pressure on the environment. The sign of ${\gamma }_{3}$ can be negative, positive or have no significant effect on environmental indicators. This may vary depending on the share of renewable energy consumption within total energy consumption in the country. If the share of renewable energy consumption is minimal, it may not have a positive or significant impact on the environment (Pata 2021b). However, if renewable energy consumption has a high share in total energy consumption, then its impact may be negative. Individuals with high human capital can be more sensitive towards environmental issues. This can reduce the negative pressure on the environment. Therefore, the sign of ${\gamma }_{4}$ is expected to be negative. The expected signs mentioned above are valid when using environmental pollution indicators such as CO_2_ emission and ecological footprint. If the environmental pollution indicator is load capacity factor, coefficient interpretations should be carried out as the inverse of CO_2_ emission and ecological footprint.

### Augmented ARDL

Pesaran et al. (2001) developed a procedure that makes it possible to investigate a long-run relationship between the relevant variables regardless of the order of integration of the independent variables *(except I(2))*, provided that the order of integration of the dependent variable is I(1). This is called the ARDL procedure.

The ARDL approach based on the unrestricted error correction model for the variables used in the present study is as shown in Eq. (2):

2 $$\begin{array}{c}\Delta lnLCF={\eta }_{0}+{\psi }_{1}\sum_{i=1}^{x}\Delta {\text{ln}}LC{F}_{t-i}+{\psi }_{2}\sum_{i=1}^{y}\Delta {\text{ln}}{Y}_{t-i}+{\psi }_{3}\sum_{i=1}^{z}\Delta {\text{ln}}NRE{C}_{t-i}+{\psi }_{4}\sum_{i=1}^{w}\Delta {\text{ln}}RE{C}_{t-i}+{\psi }_{5}\sum_{i=1}^{q}\Delta {\text{ln}}H{C}_{t-i}\\ +{\varphi }_{1}lnLC{F}_{t-1}+{\varphi }_{2}ln{Y}_{t-1}+{\varphi }_{3}lnNRE{C}_{t-1}+{\varphi }_{4}lnRE{C}_{t-1}+{\varphi }_{5}lnH{C}_{t-1}+{\mu }_{t}\end{array}$$

here; ${\eta }_{0}$= constant term, ${\psi }_{1}$, ${\psi }_{2}$, ${\psi }_{3}$, ${\psi }_{4}$, ${\psi }_{5}$= short-run coefficients, ${\varphi }_{1}$, ${\varphi }_{2}$, ${\varphi }_{3}$, ${\varphi }_{4}$,${\varphi }_{5}$= long-run coefficients, x, y, z, w, q = lag lengths while ${\mu }_{t}$ indicates the error term. In the ARDL approach, F- and t-statistics are used to investigate whether there is a long-run relationship between the relevant variables. The F-statistic represents the statistic for all variables used in the model, while the t-statistic represents the statistic for the one-period lagged value of the dependent variable. Hypotheses for F- and t-statistics are as follows:

3 $$\begin{array}{ccc}{F}_{overall}& {\text{for}}& {H}_{0}:{\varphi }_{1}={\varphi }_{2}={\varphi }_{3}={\varphi }_{4}={\varphi }_{5}=0\end{array}$$

4 $$\begin{array}{ccc}{t}_{DV}& {\text{for}}& {H}_{0}:{\varphi }_{1}=0\end{array}$$

In order to obtain a long-run relationship between the variables, both F- and t-statistics should be greater than the relevant upper bound, i.e. I(1). In recent years, the ARDL approach has been subjected to some criticism. The first point of criticism is that researchers use the F-statistic but not the t-statistic when determining the long-run relationship. Not using the relevant statistic may result in a false cointegration relationship. Moreover, the absence of a statistic for the independent variables may lead to a similar problem (Pata 2021b). Also, most of the studies using the ARDL approach do not consider the condition that the dependent variable is I(1). Based on such criticism, McNown et al. (2018) and Sam et al. (2019) modified the classical ARDL approach. This modified approach is called AARDL method. This approach is superior to the ARDL approach in that it has a statistic for the independent variables that can be used even if the dependent variable is I(0). The null hypothesis for the F-statistic developed by McNown et al. (2018) and tabulated and presented by Sam et al. (2019) for the independent variables is as shown in Eq. 5.

5 $$\begin{array}{ccc}{F}_{IDV}& {\text{for}}& {H}_{0}:{\varphi }_{2}={\varphi }_{3}={\varphi }_{4}={\varphi }_{5}=0\end{array}$$

**Fig. 3:**
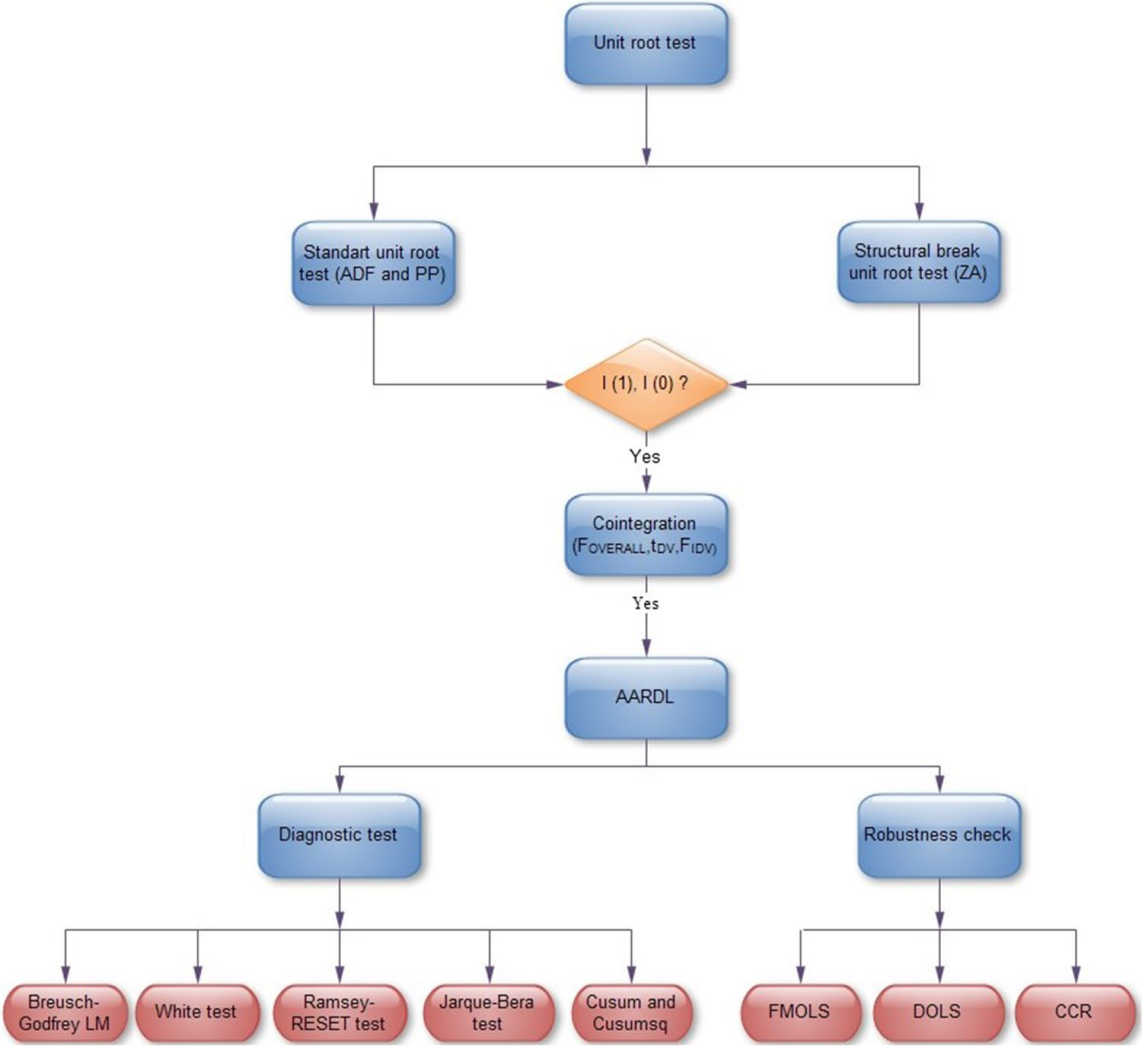
Methodological framework

In the AARDL procedure, for a long-run relationship to exist, it is required for all of the statistics shown in Eqs. (3), (4) and (5) to be significant. If t_DV_ is insignificant, degenerate case #1 emerges, and if F_IDV_ is insignificant, degenerate case #2 emerges (2018). The analysis method used in the study is shown in Fig. 3 as a diagram.

## Empirical results and discussion

The analysis section of the study started with unit root tests. First, unit root tests were performed by means of ADF (Dickey and Fuller 1979) and PP (Phillips and Perron 1988). The ADF and PP test results shown in Table 2 indicate that all variables are I(1). Second, the ZA (Zivot and Andrews 1992) unit root test that accounts for structural breaks was performed. The ZA results in Table 3 show that lnY is I(0) and lnREC is I(0) in the model with constant and trend, while all variables are I(1) in the other cases. According to the results of the three unit root tests utilized, none of the variables are I(2). Therefore, the AARDL approach can be used.

**Table 2:** Unit root tests summary

Variables	ADF (level)		ADF (1st diff.)		PP (level)		PP (1st diff.)	
	t-stat	p-value	t-stat	p-value	t-stat	p-value	t-stat	p-value
lnLCF	-2.376	0.387	-6.424***	0.000	-2.441	0.355	-6.399***	0.000
lnY	-2.111	0.527	-5.812***	0.000	-1.858	0.661	-5.985***	0.000
lnNREC	-3.045	0.132	-5.519***	0.000	-3.021	0.136	-5.519***	0.000
lnREC	-1.792	0.694	-7.887***	0.000	-1.792	0.695	-7.982***	0.000
lnHC	-3.091	0.119	-2.002***	0.000	-3.626**	0.037	-	-

1% and 5% significance level are highlighted by (***) and (**) sequentially. Optimal lag lengths are selected by SIC. The maximum lag length set at 10 using the Schwert’s (1989) approach

**Table 3:** Zivot-Andrew (ZA) unit root test

Variables	ZA_I_	ZA_T_	ZA_B_
lnLCF	-4.132	-3.205	-3.526
	*2008*	*2003*	*1997*
lnY	-5.102**	-4.964***	-5.106**
	*2009*	*2005*	*2009*
lnNREC	-3.952	-3.365	-3.656
	*2008*	*2003*	*1993*
lnREC	-3.561	-4.294	-5.187**
	*2009*	*2006*	*2000*
lnHC	-3.749	-3.629	-3.598
	*2010*	*2007*	*2010*

ZA_I_, ZA_T_ and ZA_B_ are an intercept, trend, trend and intercept with the trend of ZA estimates, respectively. 1% and 5% significance level are highlighted by (***) and (**) sequentially

Third, in order to use the AARDL approach, the presence of a co-integrated relationship was tested. In Table 4, all three statistical values are greater in absolute value compared to the upper critical *(I(1))* values at significance levels of 1% and 5%, confirming the existence of a co-integration relationship between the variables.

**Table 4:** Cointegration results of AARDL

Model (1,0,0,0,0)			Cv. 1%		Cv. 5%		Sources
			I(0)	I(1)	I(0)	I(1)	
	F_overall_	21.099	4.244	5.726	3.068	4.334	Narayan (2005)
	t_dependent_	-9.238	-3.43	-4.6	-2.86	-3.99	Pesaran et al. (2001)
	F_independent_	20.723	3.75	6.19	2.55	4.51	Sam et al. (2019)

The optimum lag length is automatically selected according to SIC

After obtaining a co-integrated relationship between the variables, in the fourth stage of the analysis, long-run elasticities were obtained and presented in Table 5. According to this, in the long run, a 1% increase in lnY decreases lnLCF by approximately 0.47%, i.e., deteriorates environmental quality. This unsurprising result regarding the relationship between economic growth and environmental quality is in line with Al-mulali et al. (2015), Mrabet and Alsamara (2017), Fareed et al. (2021), Pata (2021c) and Agila et al. (2022). Similarly, lnNREC has a negative impact on LCF. This conclusion is supported by the findings of Dogan and Ozturk (2017), Chen et al. (2019), Fareed et al. (2021), Awosusi et al. (2022) and Karaaslan and Çamkaya (2022).

**Table 5:** Long-run AARDL estimation results

Variables		Coef	Std. error	t-stat	Prob
C		13.27104***	1.240830	10.69530	0.0000
lnY		-0.467426***	0.050709	-9.217890	0.0000
lnNREC		-0.927363***	0.057968	-15.99784	0.0000
lnREC		0.034657	0.023486	1.475612	0.1467
lnHC		0.564528**	0.216446	2.608169	0.0122
Diagnostic tests					
J-B	0.053 [0.973]				
WHITE	24.399 [0.142]				
LM	3.382 [0.184]				
RESET	0.051 [0.821]				
CUSUM	Stable				
CUSUMSQ	Stable				

[] are prob values. 1% and 5% significance level are highlighted by (***) and (**) sequentially

Also, surprisingly, lnREC has a positive but statistically insignificant effect on lnLCF in the long run. This result contradicts the findings of Zoundi (2017), Mahmood et al. (2019), Danish et al. (2020) and Shang et al. (2022). The findings of the present study are in parallel with the studies conducted by Menyah and Wolde-Rufael (2010) and Baek (2016) for the U.S, Al-Mulali et al. (2015) for Vietnam, Khoshnevis Yazdi and Shakouri (2018) for Germany, Pata (2018) for Turkey, Nathaniel and Khan (2020) for ASEAN countries, Pata and Caglar (2021) for China, Çakmak and Acar (2022) for the top 8 countries in terms of oil production, and Pata and Samour (2022) for France.

Finally, it is observed that a 1% increase in lnHC increases lnLCF by approximately 0.56%, i.e., positively affecting environmental quality. This conclusion is in line with the findings of Bano et al. (2018), Yao et al. (2020), Nathaniel et al. (2021), Pata et al. (2021), Pata and Isik (2021) and Jahanger (2022).

In the AARDL method employed in the present study, a number of diagnostic tests were performed to investigate the consistency of the long-run coefficients and the properties of the error term. The Jarque–Bera, White and LM results shown in Table 5 indicate that the error term is normally distributed and free from the problems of variance and autocorrelation, respectively. Additionally, the Ramsey reset test confirms that there is no model fitting error. Finally, the CUSUM and CUSUMQ test results show that there is no structural change in the AARDL model and that it is a stable model (see Appendix Fig. 4B).

In the final stage of the analysis, the FMOLS, DOLS and CCR methods were employed to test the robustness of the long-run coefficient estimates. Table 6 shows the long-run coefficient estimation results obtained from these three estimation methods. Similar to the AARDL estimation results, the FMOLS, DOLS and CCR methods confirm that in the long run, lnY and lnNREC have a negative effect on LCF while lnHC has a positive effect and lnREC has a positive but statistically insignificant effect. The fact that the AARDL estimation results show similar findings with the FMOLS, DOLS and CCR estimation results is another indication that the method employed (AARDL) obtained consistent results.

**Table 6:** Robustness check results

Variables	FMOLS		DOLS		CCR	
	Coef	Prob	Coef	Prob	Coef	Prob
C	14.09631***	0.0000	13.71451***	0.0000	13.97524***	0.0000
lnY	-0.471282***	0.0000	-0.462773***	0.0000	-0.472667***	0.0000
lnNREC	-0.950509***	0.0000	-0.924700***	0.0000	-0.940275***	0.0000
lnREC	0.039844	0.1030	0.035370	0.1583	0.035370	0.1583
lnHC	0.535217***	0.000	0.541123**	0.0152	0.547932***	0.0103

1% and 5% significance level are highlighted by (***) and (**) sequentially

## Discussion

When the long-run coefficients obtained from our study are analyzed, it is seen that economic growth has a negative effect on environmental quality in the US. In other words, the increase in economic growth deteriorates environmental quality by reducing the load capacity factor. This implies that the developments in the main sectors that constitute economic growth in the U.S. increase energy consumption with increasing economic growth and thus accelerate the deterioration of environmental quality. In general, since fossil fuels are the main input for industry, the expansion of economic growth leads to more fossil fuel consumption, which deteriorates environmental quality (Adebayo 2022). Specifically, as noted by Kartal et al. (2023), the main input of energy consumption in the U.S. is oil. Hence, increasing economic growth increases the demand for oil, which in turn increases environmental degradation. Similarly, empirical findings show that increases in non-renewable energy sources reduce load capacity, i.e. negatively affect environmental quality. Moreover, the findings confirm that the negative impact of non-renewable energy consumption on the load capacity factor is greater than that of economic growth. This implies that the U.S. is dependent on traditional energy sources such as oil and coal for energy consumption. Indeed, as shown in Appendix Fig. 4, the U.S. meets the vast majority of its total final energy consumption from fossil resources. In this context, it is not surprising that the predominant consumption of fossil (non-renewable) resources leads to increased environmental degradation.

When the effect of non-renewable energy consumption on the load capacity factor is analysed, it is found that the effect is positive but not statistically significant. In other words, renewable energy consumption has a positive but not statistically significant effect on environmental quality in the U.S. In the U.S., the share of energy from renewable energy sources in total final energy consumption is very low compared to that from fossil sources (see Appendix Fig. 4A). Therefore, since the consumption of renewable energy in the U.S. is quite low compared to fossil sources, it can be said that it is not sufficient to improve environmental quality.

Finally, the findings reveal that an increase in human capital increases the load capacity factor. This suggests that in the long run, environmental quality can be improved by investing in human capital. Further increasing the educated population in the U.S. can create a society that is more skilful, more productive and more sensitive to environmental issues. Moreover, a skilful and productive society can play an important role in the production of renewable technologies. In this context, greater use of renewable technologies in everyday life can reduce the use of fossil-fuelled technologies. Moreover, with the presence of educated human capital, it may become easier to use resources more efficiently and save energy by preventing the unconscious use of natural resources. As a result of these situations, environmental degradation can be slowed down and even environmental quality can be ensured.

## Conclusions

The present study aims to investigate the long-run impact of economic growth, non-renewable energy consumption, renewable energy consumption and human capital on LCF in the U.S. With the aim of contributing to the literature on environmental economics, annual time series data from the U.S. for the period from 1965 to 2018 were obtained. For the estimation of the long-run effect, the AARDL approach as well as the FMOLS, DOLS and CCR procedures were utilized. The main findings of the present study are as follows.

The AARDL results obtained a cointegration relationship between economic growth, non-renewable energy consumption, renewable energy consumption and human capital and LCF. The long-run elasticities obtained from AARDL show that economic growth and non-renewable energy consumption have a negative impact on LCF, i.e., they have a deteriorating effect on environmental quality. In contrast to the negative effects, human capital has a positive and statistically significant effect on LCF. In other words, human capital has an improving effect on environmental quality. Similarly, the impact of renewable energy consumption on LCF is positive, but not statistically significant. The findings from the FMOLS, DOLS and CCR estimators used to test for robustness also support the AARDL findings.

The results of this study offer important policy implications for the U.S. First, it is important to note that the U.S economic growth reduces environmental quality by reducing LCF. To overcome this problem, the U.S government should use the additional revenues from its economic growth as resources to improve environmental quality. These resources should be used for the development of energy-saving technologies, eco-friendly clean energy sources, and technologies to reduce fossil fuel dependence for economic value-added sectors. In this way, the negative pressure of economic growth on the LCF can be mitigated. Otherwise, an economic growth strategy that postpones environmental quality will progressively reduce the U.S LCF, making environmental conditions unsustainable and setting the stage for irreversible environmental consequences.

Secondly, the U.S. should reduce its intensive use of fossil resources to establish a sustainable environment and combat climate change. This can be done by providing incentives for fossil-intensive industries to transition to renewable technologies. Additionally, policies should be developed to ensure energy efficiency, which is important in combating environmental pollution. For example, the government should support energy efficiency workers and R&D on the efficient use of energy. Policymakers should develop policies to encourage the use of environmentally friendly machinery and equipment in industrial sectors and transportation. Finally, legislation (e.g., a carbon tax) should be enacted at the national and state level to reduce the use of fossil fuels. Implementation of these policies would help improve environmental quality in the U.S.

As another policy strategy, the U.S. government should review its existing renewable energy policies. This is because the current use of renewable energy in the U.S. is not sufficient to improve environmental quality. Increased use of renewable energy can reduce pressure on the ecological footprint. Furthermore, an increase in the use of renewable resources can reduce the demand for non-eco-friendly resources. This could result in increased biocapacity. As a joint result of these two situations, the load capacity factor and, consequently, environmental quality may increase. In this context, the U.S. government should invest in increasing the share of renewable energy consumption within final energy consumption. For this purpose, various incentives should be implemented to pave the way for the use of environmentally friendly renewable energy. For example, tax exemptions can be provided to encourage the use of renewable energy. Subsidies should also be provided to support R&D expenditures for industries seeking to develop renewable technology.

As a third and final strategy, the U.S should invest more in human capital. The power of human capital can build a constituency in the U.S that demands wider adoption of eco-friendly technologies. This constituency could force polluting industries operating across the country to adopt renewable technologies. Moreover, this constituency can also play a role in raising environmental awareness. For example, the use of eco-friendly technologies can help raise awareness about energy efficiency, recycling, and a sustainable environment. To this end, the U.S. government should develop, or improve upon, documents on the causes of environmental degradation and what is necessary and essential to prevent it. This document should be integrated into all U.S educational curricula. The U.S government can therefore reduce the negative pressure on the environment by raising environmental awareness. As a result, environmental quality can improve.

The present study offers several opportunities for researchers interested in conducting studies on environmental economics. The load capacity factor, which is the main focus of this study, is a new and comprehensive environmental indicator. Therefore, in future studies, various factors that have an impact on the environment such as the export diversification index, trade openness, and urbanization can be examined both on the basis of a single country using time series techniques and on various groups of developed and developing countries using panel data techniques.

## Figures and Tables

**Fig. 4:**
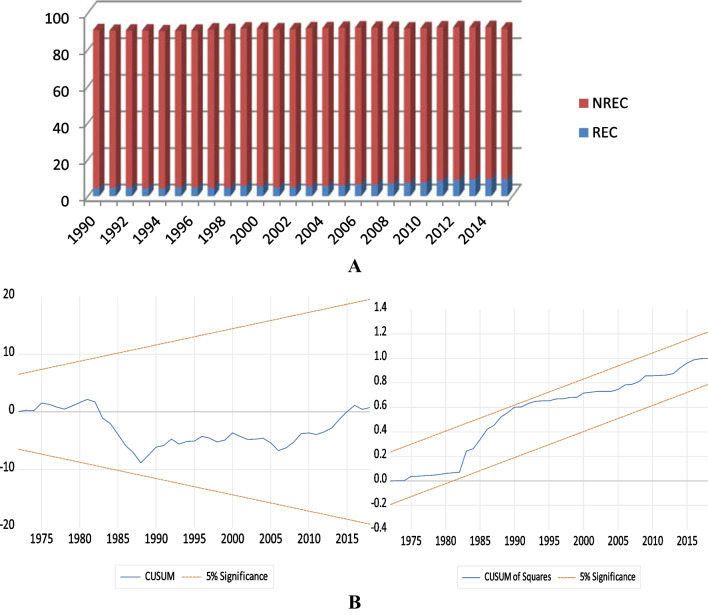
**A**. The share of fossil fuels and renewables in total final energy consumption in USA. Source. World Bank, (2022). **B**. The results of the CUSUM and CUSUMSQ

## Data Availability

The analyzed during the current study, LCF were obtained from Global Footprint Network Indicators (GFN). Y were obtained from World Bank Indicators (WDI). NREC and REC data were obtained from Our World Data (OWD). HC were obtained from HC data from Penn World Tables version 10.0 developed by Feenstra et al. (2015).

## Appendix

See Fig. 4
